# Allyship in Healthcare for People With Learning Disabilities as a Praxis of Respect, Attention and Collaborative Action

**DOI:** 10.1111/1467-9566.70047

**Published:** 2025-05-14

**Authors:** Bojana Daw Srdanovic

**Affiliations:** ^1^ Peninsula Medical School University of Plymouth Plymouth England; ^2^ Cornwall Intellectual Disability Equitable Research (CIDER) Cornwall Partnership NHS Foundation Trust Truro England

**Keywords:** allyship, disability allyship, disability allyship in healthcare, intellectual disability, learning disability

## Abstract

There is a dearth of literature focusing on how allyship in health may be enacted in relation to people with learning disabilities (LD). This is concerning, because people with LD are vulnerable to health inequalities and forms of medical dehumanisation including do‐not‐resuscitate orders, diagnostic overshadowing and overprescription of psychotropic drugs. Deploying critical disability studies as a lens through which to understand disability, this paper reviews models of disability allyship developed in healthcare, research and theatre. In doing so it advocates transformative allyship as a model that can both animate action in support of people with learning disabilities and accommodate the involvement of others, including clinicians, carers and relatives, without compromising the all‐important commitment to supporting disability cultures. The paper presents and analyses ethnographic data gained through observations of eleven healthcare appointments between seven clinicians and five patients with LD, undertaken as part of the ESRC‐funded study Humanising Healthcare. It documents the potential of transformative allyship in healthcare to transform harmful disablist practices through emphasising respect, attention and collaborative action while also noting that broader structural conditions and diagnostic technologies limit the extent to which clinicians can enact transformative allyship.

## 1 Introduction

Because of multiple terms used to describe learning disabilities, it is necessary to begin this paper with a note on language. The term ‘learning disabilities’ is used in the UK and preferred by the researchers with learning disabilities who are working on Humanising Healthcare. Other countries use labels such as ‘intellectual disabilities’, ‘development disabilities’, and ‘cognitive impairments’. I refrain from offering an administrative definition of learning disabilities—which would normally refer to individual issues of competence and difficulties in accessing, processing and/or articulating information—in response to the wider aims of this paper to centre people thus‐labelled as a marginalised social group.

People with learning disabilities occupy a complex position in healthcare (MacLeod and MacLure 2020): they experience a potentially higher risks of congenital and acquired health conditions, including epilepsy (Shankar et al. 2019), mental health conditions (Adams 2015; Choi et al. 2020) and diabetes (McVilly et al. 2014), and are affected by health inequalities arising from a number of factors. This paper uses Critical Disability Studies (CDS) as a lens through which to understand these inequalities. Being a ‘field of scholarship and activism’ (Goodley 2017) CDS emphasises the views and experiences of disabled people and seeks to scrutinise ‘not bodily or mental impairments but the social norms that define particular attributes as impairments’ (Minich 2016). Critiquing the disability/impairment split central to the social model, CDS emphasises the sociocultural production of both disability and impairment (Shildrick 2020)—a shift that has been instrumental to extending the social model to include learning disabilities (Goodley 2001). CDS's critique of solely materialist accounts has opened space for dialogue between disability studies and medical sociology, fostering capacious accounts that eschew neither structural oppression nor impairment effects (G. M. Thomas 2022).

CDS regards disability not as an individual pathology but as a complex social, political, cultural and economic phenomenon, and a social identity grounded in disabled people's shared experience of disablism and ableist oppression. Disablism refers to practices that oppress disabled people (C. Thomas 2007), whereas ableism is a broader term describing the deeply embedded tendency to value ‘able bodies’ (Wolbring 2008). Providing an environment ‘for disablism to grow’ (Goodley 2017, 57), ableism underlies various forms of disability stigmatisation, including stereotyping, discrimination and segregation (Nario‐Redmond 2019). Consequently, CDS draw attention to the ways in which disablism and ableism produce the many inequalities, including those in health, that disabled people experience. For example, clinicians' failure to provide reasonable—and legally ratified—accommodation to disabled people (Iezzoni et al. 2022); stigma's effects on clinicians' care for disabled people (Mello et al. 2020) and disabled people's access to and in healthcare (Carew et al. 2024); the effects of internalised disablism on the well‐being of disabled adults (Reeve 2012) and children (Cooper 2020). Disabled people and their allies have long campaigned to challenge both disablism and ableism. Despite this, there is a notable lack of literature focusing on learning disability allyship in healthcare.

Drawing on ethnographic data from the study Humanising Healthcare, this paper seeks to address this gap. Through describing and analysing how clinicians enact allyship in relation to patients with learning disabilities, this paper focuses on the praxis of learning disability allyship in healthcare specifically. After introducing the study Humanising Healthcare under the following subheading, I turn to theorising allyship. In order to approach, understand and explicate this concept, I engage with social psychology literature on general, anti‐racist and disability allyship. I then turn to disability allyship in healthcare, noting that the majority of literature on learning disability allyship specifically is located in the area of inclusive research. Drawing on those sources, I highlight the need for a model of allyship that responds to the fact that the autonomy of people with learning disabilities is routinely circumscribed by non‐learning‐disabled others. I finally turn to transformative allyship as a suitably nuanced model that has been developed in theatre, a field that has since at least the 1980s (Clements 2017; Tomlinson 1982) engaged with disabled people as artistic collaborators, often with an emancipatory/transformative agenda.

Following Section 3 and its focus on the theory of allyship, I introduce the paper's methodology in Section 4 before presenting, in Section 5, four snapshots from Humanising Healthcare's ethnographic fieldwork, which illustrate how clinicians act as allies of people with learning disabilities and what challenges they encounter in doing so.

## 2 A Study: Humanising Healthcare

Humanising Healthcare is an ESRC (Economic and Social Research Council)‐funded project that documents and explores what compassionate, caring, enabling and responsive healthcare looks like for people with learning disabilities. Humanising Healthcare is co‐produced with researchers with learning disabilities who are skilled and experienced researchers and self‐advocates. They work together with university‐based researchers in designing the study and methods, gaining funding, producing resources and outputs, theorising humanising healthcare and analysing the data. The project began in September 2022 and will run until August 2025, across four phases—literature review, co‐producing theory, co‐designing methods, implementing fieldwork and co‐producing findings and analysis.

The qualitative data for the project are collected by two research associates, Bojana Daw Srdanovic and Nikita Hayden, across two sites: a learning disability service in the Southwest of England and a neurology service in Wales. Data are collected through ethnographic observations of appointments between clinicians and patients with learning disabilities, and narrative interviews with those same patients and their close others, including friends, partners, carers and parents. As described in more detail in Section 4, the initial analysis of fieldnotes led me to posit that some of the observed humanising practices could be understood as forms of learning disability allyship.

## 3 Allyship, Disability and Healthcare

Despite having gained much traction in both academic and popular contexts, allyship remains an ambiguous concept, partly due to being theorised across several bodies of distinct literature (De Souza and Schmader 2024). Although there is broad agreement that allyship involves actions members of advantaged groups undertake in support of disadvantaged groups (De Souza and Schmader 2024; Kutlaca et al. 2020; Radke et al. 2020), not all authors insist on action as a necessary component. For example, having researched the qualities people of colour perceive in white allies, Brown and Ostrove (2013) identify two categories: affirmation, which denotes a commitment to fostering respectful and caring attitudes towards members of disadvantaged groups, and informed action, which includes understanding racial difference and racism and taking action to address it. They suggest that allies may be distinguished into ‘friends’ who may be ‘high on affirmation but not on informed action’ and ‘activist’ who may be ‘an informed actor but not necessarily affirming’ (Brown and Ostrove 2013, 2220). Similarly diverging from action as a defining feature of allyship, some authors focus on motivation, arguing that because allyship needs to be motivated by broadly egalitarian ideals and a desire to end oppression, members of advantaged groups who engage in allyship action to benefit themselves or their social group cannot be called allies (Radke et al. 2020, 293). Paying attention to both action and motivation, the term ‘performative allyship’ has emerged to describe ‘easy and costless actions’ which fail to ‘challenge the status quo’, are motivated by personal gain, and may in fact harm members of both advantaged and disadvantaged groups (Kutlaca and Radke 2023).

De Souza and Schmader (2024, 3) argue against ‘identity‐oriented’ definitions of allyship, which emphasise behaviours and motivations in designating allies. Instead, they take a ‘practice‐oriented’ approach, defining allyship as a

custom (a) comprised of several actions rather than one action alone taken by advantaged group individuals, which are (b) motivated by egalitarian goals to support outgroups that are disadvantaged and are (c) recognized as supportive by those they seek to support.

(De Souza and Schmader 2024, 4)

De Souza's and Schmader's approach is well‐suited for disability allyship in healthcare as it is both conducive to praxis and congruent with the findings of Ostrove and colleagues who, having studied disabled people's perceptions of non‐disabled allies, similarly stress the importance of chronic engagement (Ostrove et al. 2019). De Souza and Schmader’s (2024) point that allyship must be recognised as supportive by its target is similarly repeated as Ostrove and colleagues document that disabled people appreciate as allies those non‐disabled others who offer appropriate and solicited help, rather than assuming incompetence (Ostrove et al. 2019, 931). In this account, allies stand out as understanding individual access needs and respecting reasonable adjustments without treating disabled people differently in other areas (Ostrove et al. 2019, 932–933). Indeed, the understanding of access needs emerges as a matter of connection, even intimacy (Ostrove et al. 2019, 933). This need for allies to both understand the social aspects of disablement and gain deep understanding of the individual is repeated in other research where informants describe allies as people who are

devoid of disability prejudice but who know and despise it when they see it […] make the effort to learn who their disabled associates are in their full glory and their full ordinariness. In disability cultural parlance, these nondisabled friends and relatives are the ones who ‘get it’.

(Gill 2001, 368)

This dual focus on the social and the individual highlights that, despite being united by the shared experience of ableist oppression, disabled people are a heterogenous group whose access needs fluctuate across the group, and even on an individual day‐to‐day basis. Indeed, not everyone who may be classified as disabled self‐identifies as such or engages with disability rights (Beart et al. 2005; Bogart et al. 2017; Price and Goyal 2016). Nevertheless, disability allyship remains relevant and potentially beneficial as disidentification with disability status offers limited protections against the structural and interpersonal harms of ableism and disablism which disability allyship seeks to redress.

There are similarities between anti‐ableist and anti‐racist allyship: both disabled people and people of colour identify political and social aspects of allyship, and emphasise allies' ability to connect, be respectful and non‐judgemental (Ostrove et al. 2019). However, although disabled people experience similar issues as people of colour and other marginalised groups, they are also affected by unique challenges surrounding ‘physical barriers and gaps in accessibility of communication and information’ which play out as barriers to healthcare (Swenor 2021, 359). Disability allyship in healthcare poses some specific challenges as a result of the deep‐rooted tension between disability and medicine which is broadly focused on ‘prevention and treatment of disability’ (Swenor 2021, 359). As Swenor stresses, although the prevention and treatment of illness is ‘important and necessary’, the ‘myopic focus’ on disability as the ‘antithesis of health […] perpetuates stigma, ableism, and inequities for the disability community’ (Swenor 2021, 359).

Despite these challenges, there is a growing body of scholarship documenting the significance and scope of anti‐ableist allyship in healthcare as a set of actions in support of disabled people. Meeks and colleagues (2018) highlight the need for more clinicians with lived experience of disability. In accordance with the social model of disability (Barnes 2013; Oliver 1990)—itself a precondition to any form of disability allyship (Evans et al. 2005)—disability allyship in healthcare seeks solutions not to effects of disadvantage but to the systems that create it (Nixon 2019). As disabled people may also need and value rehabilitation (Shakespeare 2014, 6), disability allyship in healthcare can take the form of resisting ableist perspectives that frame rehabilitation as ‘fixing’ and the non‐disabled clinician as a saviour, rather than ‘collaborative partner’ (Feldner et al. 2022). Thus, disability allyship in healthcare includes

amplification of disabled voices and experiences in all aspects of society, meaningful engagement within disability communities in solidarity, engaging in inclusive practices, rejecting performative allyship, rejecting harmful disability narratives, becoming educated about disability oppression, and leveraging privileged positionalities to call out ableism and injustice and produce actionable change.

(Feldner et al. 2022, 3)

Arguing that allied clinicians can improve disabled people's health outcomes by supporting their construction of a disability identity, Forber‐Pratt and colleagues (2019, 126) produce a strikingly similar list of ways to ‘show up’ as an ally:

Understand intersectionality; ask and respect choice of terminology; embrace principles of individual design; act as an ally; recognise inspiration porn and oversensationalizing disabled people; be aware of the current disability rights issues facing the community; check internal disability‐related biases; embrace cross‐cultural disability solidarity.

(Forber‐Pratt et al. 2019, 126)

Consistent with the emphasis on self‐education as a core principle of disability allyship (Evans et al. 2005; Forber‐Pratt et al. 2019), allyship emerges as a promising approach to improve disabled people's healthcare through addressing clinicians' limited knowledge regarding their legal duties to provide reasonable accommodation to disabled patients (Iezzoni et al. 2022). Moreover, a commitment to allyship can affect clinicians' biases which, as observed during the COVID‐19 pandemic, can produce stereotypical quality‐of‐life judgements leading to medical decisions that exclude disabled people and devalue their lives (Iezzoni et al. 2021, 305). Here, people with profound learning disabilities and other cognitive impairments are at greatest risk of discriminatory practice (Mello et al. 2020).

Swenor (2021) points to health research as an arena for the enactment of disability allyship, highlighting an absence of both disability‐related data and disabled researchers. Issues surrounding research are repeated across literatures as disabled people have been historically exploited as research subjects (Oliver 1999), under‐consulted (C. Thomas 1999, 152), and over‐researched (Hope 2019; Kaehne and O’Connell 2010) with research often being used to manage and control them rather than to ‘achieve social justice’ (Ostrove et al. 2019, 929). Such exploitation engendered emancipatory (Barnes 2004) and inclusive (Walmsley and Johnson 2003) research, creating conditions for disability allyship in research where much of the literature on learning disability allyship specifically is produced.

The literature surrounding learning disability allyship in research suggests a concern with relationships, and the inclusion and representation of the perspectives of people with learning disabilities. To illustrate, allies are instructed to:

(1) Learn from and nurture long‐term, mutual relationships with people with ID; (2) Amplify the voices of people with ID in institutional structures that influence research; (3) Infuse anti‐ableist frameworks into our own research; and (4) Embody a career‐long commitment to disability rights, reflexive practice, and growth.

(McDonald et al. 2023, 399)

Milner and Frawley (2019, 385) highlight that allied researchers need to be more careful to include the ‘hard to reach’ voices of people with complex communication needs. Describing narrative allyship as both a methodology for co‐authoring autobiographical accounts with people with learning disabilities and the praxis of ‘doing allyship’, Pombier (2020, 233) instructs researchers to ‘further the interests of narrators, as the narrators themselves define them; optimise the autonomy of narrators; tell stories with, instead of about, narrators’. She highlights that ‘[n]ondisabled third parties are often gatekeepers to agency for people with intellectual disability, creating power dynamics that researchers who work as allies across ability will need to understand and navigate’ (Pombier 2020, 244).

The involvement of ‘nondisabled gatekeepers to agency’ differentiates the situation of people with learning disabilities from that of most other marginalised groups and is thus of central importance for learning disability allyship. Although the denial of ‘reason’ is a sleigh of hand that has historically served to dehumanise most humans apart from white, Western, heterosexual, property‐owning men (see e.g., Schalk 2017), most marginalised groups have—at least in principle—asserted their right to autonomy. For people with learning disabilities and similar *cognitive* impairments, autonomy remains a matter of negotiation with others. Adding further complexity and reiterating the centrality of relationships in learning disability allyship, others involved in the lives of people with learning disabilities can also serve to facilitate autonomy (Goodley and Runswick‐Cole 2016), rather than nefariously guarding it as the term ‘gatekeeper’ perhaps suggests. Therefore, learning disability allyship in healthcare requires a model that centres disability rights and action while being capacious enough to accommodate the involvement of others in the delicate area of autonomy. The field of theatre and disability approaches such a model with the concept of transformative allyship.

Unlike many other contexts in which people with and without learning disabilities encounter each other, theatre emphasises both interdependence and risk. In doing so it animates pedagogies focusing on a sense of safety and interpersonal trust as a precondition to risk‐taking (Busby 2021). This markedly contrasts healthcare settings which, despite being high‐stakes environments, are interpersonally risk‐averse (McCreaddie and Payne 2014) and can therefore limit the scope for action in support of people with learning disabilities. As a result of theatre's reliance on risk‐taking, the field has developed complex practices and theories of care, particularly under the broad umbrella of applied theatre (Thompson 2009, 2023) where much work with and by theatre‐makers with learning disabilities is located. Often taking place in non‐theatrical locations, including care homes, prisons and refugee camps (H. Nicholson 2005), applied theatre is a field of creative practice and scholarship that is not only acquainted with marginalised groups but has developed complex understandings of the aesthetics and politics of collaboration (Harpin and Nicholson 2016; Kuppers 2007). Moreover, despite no longer being a necessarily political practice, applied theatre is rooted in the more overtly political milieu of community arts in 1970s and 1980s Britain (Jeffers 2017), a period when—owing to burgeoning deinstitutionalisation—people with learning disabilities began engaging with theatre in ways that emphasise emancipation and representation rather than therapy and normalisation (Calvert 2009; Conroy 2009; Tomlinson 1982). It is important to highlight that these encounters have not been politically unproblematic: collaborating non‐disabled artists can be oppressors as well as allies (Perring 2005), and theatre can both challenge and re‐produce stereotypical portrayals and social identities (Hargrave 2015). Nevertheless, in at least seeking to centre disabled people, celebrate difference and resist harmful portrayals, theatre produced with/by disabled people contrasts medicine where disability has historically been treated as, broadly, a problem (Swenor 2021).

In recent years, theatre's simultaneous focus on care and the politics of collaboration has been extended to questions of allyship (Hadley 2020; Hadley et al. 2022). Advocating ‘cultural safety, respect and trust in […] allyship relationships’ (Hadley et al. 2022, 1) one such approach explicitly builds on insights from healthcare as the concept of cultural safety was developed by the Māori nurse Irihapeti Ramsden (1993) highlighting that clinicians who belong to culturally dominant groups may alienate patients from marginalised groups. Ramsden's concept of cultural safety echoes accounts of allyship (Feldner et al. 2022; Forber‐Pratt et al. 2019) insofar as it calls on clinicians to recognise their bias and develop new, more inclusive, practices in partnership with patients. Hadley, speaking from the field of theatre, adds the call for a praxis to replace the figure of the ally as an ‘altruistic support or advocate’ with ‘transformative understandings of allyship as an interdependent social, professional and aesthetic practice, in which allies partner with disabled artists to achieve artistic outcomes, in a disability culture context’ (Hadley et al. 2022, 2). Here, disability allyship emerges not as a blueprint of practices, but as a collaborative process that emphasises safety, trust, respect and leads to transformation (Hadley 2020; Hadley et al. 2022).

Although developed in an arts context, Hadley and colleagues' (2022) model of transformative allyship can be extended to the medical context. Indeed, in emphasising interdependence and collaboration, it adds to the field's understanding of learning disability allyship in such ways that allow for the navigation of complex relationships without sacrificing the all‐important elements of considering disability culture and focusing on action. Transformative allyship, moreover, fulfils the condition of being grounded in the social model (Nixon 2019) and reiterates partnership as a recommendation made by authors writing from a healthcare perspective (Feldner et al. 2022; Forber‐Pratt et al. 2019). Being practice‐based, transformative allyship has similar strengths as De Souza and Schmader’s (2024) model but its emphasis on disability culture rather than egalitarianism is better‐suited for the learning disability context because it centres disabled people's responses to their historical and current marginalisation.

## 4 Methodology

The data underlying this paper have been gained through the ethnographic observation of medical encounters between clinicians and patients with learning disabilities within a specialised learning disability service in the UK. Ethnographic observation has been deployed to illuminate the experiences of people with learning disabilities who have cancer (Tuffrey‐Wijne et al. 2009) and those living in locked wards (Fish 2016). It is worth noting that there is a significant amount of research into learning disability and health that draws on conversation analysis, as a form of ethnomethodology (Chinn 2022; Chinn and Rudall 2021; Mander 2016; C. Nicholson et al. 2021; Pote et al. 2011).

At the time of writing, six patients with an official diagnosis of learning disabilities and nine clinicians had been recruited from within the learning disability service. The clinicians volunteered to recruit patients in their care after I introduced the project at several of the service's team meetings. Once the suggested patients had consented to participate, I sought the clinicians' consent to observe the appointments. For this paper I focussed on the observations of a total of eleven healthcare appointments involving five of the patients and seven of the clinicians. Three patients were female, two male, with ages ranging from early 20s to late 50s. All patients were white, which—being a noted shortcoming in learning disability research (Rabbitte and Adam 2023)—presents a limitation of this paper. I captured the observations in descriptive handwritten field notes (Brewer 2000). I then conducted a thematic analysis (Braun and Clarke 2006) of the fieldnotes, using a combination of inductive and deductive engagement with the data (D. R. Thomas 2006) to identify, through the analytic lens of CDS, examples of humanising healthcare practices. Using a process‐coding approach (Saldaña 2014), I produced initial codes, which I then reviewed and grouped into provisional themes. Finally, I reviewed those, arriving at three main themes:

- Treating the patient with respect (addressing the patient, not the carer; listening attentively to the patient).
- Removing disabling barriers (explaining well; noticing when the patient is not understanding; changing strategies when explaining things; using accessible information in Easy Read).
- Centring the patient as expert (taking the patient's knowledge seriously; asking the patient for their views; discussing next steps in treatment; patients training staff; patients chairing meetings).

Positing that these practices may be instances of allyship, I conducted a literature review of the concept, then analysed the fieldwork notes one more time. Informed by literature, I undertook a deductive analysis of the fieldnotes, searching for instances where clinicians were taking into account the social aspects of disablement (Evans et al. 2005; Gill 2001; Nixon 2019); acting in partnership with patients (Feldner et al. 2022; Forber‐Pratt et al. 2019), and enacting transformation in services and relationships (Hadley 2020; Hadley et al. 2022). I gathered the instances demonstrating at least one of the above described criteria and analysed them once again, extracting three specific humanising attitudes the clinician allies embody in relations to their patients:

- Respect for patients, their views, relationships, decisions and preferences
- Attention to the patient, their levels of understanding, engagement, dignity and comfort
- Commitment to collaborative action

I then describe, in four vignettes, moments that illustrate those attitudes in praxis. As ‘snapshots, seeking to capture a fleeting moment’ (Bloom‐Christen and Grunow 2022, 5) the vignettes retain the necessary contextual information while focusing closely on the enactment of allyship. Each vignette is followed by my analysis which, following Snow and colleagues (2003), seeks to link the vignettes to previously presented theories of allyship, particularly so allyship as a transformative practice.

## 5 Four Moments of Allyship in Healthcare

In presenting moments of learning disability allyship in healthcare, I begin with two vignettes that illustrate collaboratively developed transformations of relationships between patients and healthcare professionals. I then present a vignette that spotlights attentive presence as a subtle but deeply transformative act of allyship. Finally, I describe a moment that highlights the limits of individual allyship in healthcare. Allowing for a nuanced critique of both structural oppression and accounts that too‐readily conflate medicine with oppression, CDS have been instrumental to identifying the moments presented below.

### 5.1 Moment One—Training

Polly is a woman with learning disabilities. She lives in a residential care home and has been referred to Anna, a dietician specialising in work with patients with learning disabilities. Polly was referred in order to get support with losing weight. Polly’s previous efforts, supported by care staff, were partially effective insofar as she was losing weight. This success was, however, limited by the negative effects dieting had on Polly’s broader wellbeing, making her anxious and hyper‐focussed on her weight. I learn from conversations with Polly that she was motivated into dieting by her weight exacerbating existing mobility issues and causing her sleeping problems and low mood, which were likely related not only to her weight but also the anxiety that she was at risk of developing diabetes.

After being referred to the dietician, Polly began to lose weight steadily and slowly, that is at a rate recommended as healthy and sustainable. Importantly, her mental health also improved. The dietician, Anna, interprets this as an effect of Polly’s changing habits. First, she has been urged to weigh herself only once a month; second, Anna’s dietary advice coalesces around adding rather than taking away. As a result, Anna suggests, Polly is less focussed on the things she should not consume, but rather on the things that she has gained in her diet. Conversations with Polly reveal gains in excess of the familiar healthy foods: long walks which she is increasingly undertaking with other residents who wish to exercise more; deepening relationships with animals she takes on walks and as whose main carer she now identifies; pleasure in her body as it moves with less pain; joy at new clothes she is buying. Finally, there is also Polly’s changing status in the care home as she emerges as an expert not only on her own health but on weight loss more broadly. Polly’s new identity as an expert in weight loss and nutrition has been slowly developing as others, including staff, ask her for weight loss advice. It, however, reaches a new point when Polly delivers training to staff, an intervention that she has developed in partnership with Anna.

The training has a pragmatic aspect insofar as staff members get an opportunity to familiarise themselves with Polly's diet and learn how to support her more adequately. Thus, the training not only facilitates the emergence of Polly as an expert on her health and care needs but also positions Anna and the care staff as partners in Polly's realisation of her care needs—a relationship that Feldner and colleagues (2022) advocate as an ideal of allyship in healthcare. Importantly, as Polly's example highlights, this relationship is one of transformative allyship, insofar as this new relationship engenders a change in the roles, perhaps even the personal and social identities, of the key actors. To illustrate, the partnership with Anna has facilitated Polly's transformation into a trusted and often consulted expert on healthy eating and exercise. The respect and admiration this garners is significant as people with learning disabilities are routinely relegated to the status of ‘perpetual learners’ (Williams et al. 2009, 825) with people without learning disabilities in the roles of educators. Admittedly, Polly's transformation is rooted in learning, in this case learning about nutrition, eating plans and so on. Crucially, however, that learning is oriented towards becoming an expert in her care, finally transforming her into an educator herself. Thus, although an understanding of the patient as one of the experts appears as a precondition to partnership in healthcare, its successful realisation may have educational aspects. Put differently, patients may need to learn about health practices in order to be experts beyond simply knowing their life situations. This, in turn, highlights that the realisation of partnership in healthcare, as an important aspect of allyship, requires resources to facilitate such learning.

### 5.2 Moment Two—Teams

Josh is a man in his twenties. He has learning disabilities and lives in supported independent accommodation. He works closely with a clinical psychologist, Sam, who specialises in working with people with learning disabilities. Sam organises multidisciplinary meetings that are chaired by Josh and include his support workers, a social worker, nurse and family members. In addition to chairing the meetings, Josh sets the agenda and emails it to invitees ahead of the meeting. Although the entire team are involved in the meeting, Josh’s status as the chair is apparent in his delegating to other members of the team. At one point, care staff’s views on Josh’s personal hygiene begin to dominate the conversation. Josh steps in and asks them to stop, explaining that while he is happy to have this conversation with the relevant staff members at home, he doesn’t want to discuss his hygiene in front of the entire team. The team agree and Josh moves to the next point of the agenda.

Moment Two highlights the patient's increased control as a transformation. Although, similarly to Polly, Josh acts as a member within a team of experts, his position as chair affords him an additional level of control, most notably demonstrated when he intervenes to stop staff discussing his personal hygiene in front of a large multidisciplinary team. Contrasting the historic subjugation of people with learning disabilities to the medical gaze, that level of control not only highlights Josh as an agent in his healthcare but also permits him to protect his privacy and dignity. In doing so Josh asserts his agency as an aspects of humanity that is routinely denied to people with learning disabilities. Indeed, even the apparently mundane act of Josh, like Polly, being present during a team meeting can produce a humanising effect insofar as ‘care’ conversations behind the backs of people with learning disabilities can take a notably dehumanising tone (Goodley and Runswick‐Cole, forthcoming).

Moment Two is thus an instance of allyship that transforms relationships, but also an example of allies partnering with disabled people in a disability culture context (Hadley et al. 2022). To illustrate, Josh's team enacts its practice in contrast to the harmful disablist and dehumanising practices that have historically marked the relationships between medical professionals and patients with learning disabilities. In doing so it changes the aesthetics of a multidisciplinary team meeting: thanks to it being chaired by a patient with learning disabilities, the tone of the discussions is conversational and attentive, rather than laden with objectifying jargon as might otherwise be the case; the agenda is presented in the words of a man with learning disabilities, using his own version of Easy Read. This aesthetic change has the effect of the multidisciplinary meeting becoming infused with some of the practices, values and symbols of disability culture: a resistance to the injustices of the past, Easy Read materials, plain English, attentiveness and inclusive conversation as conditions for dis/autonomy (Goodley and Runswick‐Cole 2016).

Gesturing towards the limits of allyship, Moment Two illuminates the extraordinary positions of people with learning disabilities: although personal hygiene may of course be seen as a proxy to Josh's mental health, there is also a distinct feeling that the team's insistence on Josh conforming to conventional views on hygiene is an instance of people whom the person with learning disabilities regards as allies acting as gatekeepers of acceptability and normality (Clifton et al. 2020, 143). Such readings require careful contextualising because apparent efforts to control and surveil may not be driven by gratuitous paternalism but a genuine need to safeguard a person with learning disabilities from bullying and hate crime. Put differently, what might be dismissed a phase of rebellious nonconformity in a young person without learning disabilities, becomes potentially perilous non‐normativity in their peers with learning disabilities. Here, the extent to which clinicians can enact allyship is limited by broader social conditions such as the risk of a young person with learning disabilities being further stigmatised lest they successfully conform to social norms.

Moment Two, also highlights how—in healthcare—allyship as a truly transformative practice may be limited by the ontological tensions between medicine, and the emancipation of the populations it pathologises. To illustrate, although the policing of Josh's hygiene may be, as suggested above, related to stigma avoidance, such patrolling of personal care choices is also a central aspect of social care, at times figuring as a ‘duty of care’ that is often in conflict with personal choice and indeed liberty (Jingree 2015; Series 2022).

### 5.3 Moment Three—Stepping Stones

During the above described meeting, Josh expresses his dissatisfaction with the amount of support he receives. He explains that rather than living on his own and being supported by staff for most of each day, he would prefer to share a flat with friends and have fewer hours of support. Sam listens attentively to Josh’s explanation that living with friends would relieve him of sole responsibility for everything in his flat. Having listened, Sam begins to explain that the high support package is directly related to Josh not complying with expectations surrounding his care, ranging from healthy cooking to personal hygiene. It is clear that Josh does not understand what Sam is explaining. Sam notices Josh’s confusion. She asks if he understands. Josh shakes his head. Sam pauses for a moment, then gets a piece of paper and asks Josh if he knows the game *Snakes and Ladders*. Josh nods and Sam goes on to explain his situation as an instance of that game where successfully conforming to certain terms brings him closer to his goal of minimal support. Josh laughs and the atmosphere in the room relaxes. Sam smiles too. She takes another piece of paper and explains that Josh’s goal needs to be reached via stepping stones. Here, Josh becomes very animated and says that he understands now. Sam suggests that Josh and his team could draw a plan around the stepping stones. Josh agrees. When I visited Josh a few weeks later, he lived in a new flat; still on his own, but closer to the shared communal space. He had a neat new haircut and had started to cook. On his wall hung a poster showing the stepping stones towards the good life as *he* envisages it.

Exemplifying as it does a commitment to removing disabling barriers, Moment Three reveals Sam's attentive presences as a subtle act of allyship. Rather than concerning planned and coordinated alterations in practice, such as Moments One and Two do, this instance of allyship was enacted spontaneously, transforming Sam and Josh's communication and—consequently—Josh's cooperation with his care team.

Sam's response to Josh's confusion attests to a form of empathic and proactive presence that itself draws attention to interdependence as an affective, as well as sociopolitical phenomenon. The latter, which we might call macro interdependence, ensures our physical survival, and is itself so well concealed in the imaginary of the independent human that feminist philosophers have for decades insisted on its proper recognition (Kittay 2019; Tronto 1993). In addition to macro interdependence we are also enmeshed in the micro interdependencies of co‐creating each social moment in and through presence to each other.

This aspect of interdependence appears in theatre's registers of care where it is theorised as an empathic presence to the other person (Thompson 2023). To illustrate, the words we utter are, if not responses to another's words, then fashioned so that another understands them and may act on them. Theatrical improvisation necessitates and thus highlights the presence and care that is required for successful communication. The fact that in everyday life such care routinely bypasses one's awareness speaks not only of the differences between the theatrical and quotidian, but of the specific kind of privilege reserved for those fitting seamlessly into the norm. In contrast—as noted by researchers with learning disabilities working on this project—patients with learning disabilities are routinely denied the care they require to successfully process medical information. Because such carelessness in communication with people with learning disabilities is not only commonplace but an important aspect of their disablement, Sam's sensory and emotional attunement to Josh is a form of transformative practice—allyship—insofar as it renders comprehensible something as crucial as his own care plan.

Moment Three also demonstrates that the active engagement of people with learning disabilities in the planning of their care can reveal discrepancies between their individual desires and wishes, and the goals that they are being ‘supported’ to achieve. Josh's observation that he would prefer to live with others thus strikes me as an eloquent commentary on the often inadequate narratives of the ‘good life’ that are imposed on people with learning disabilities. Representing independence as it does, Josh's flat is at once the alleged holy grail of housing for people with learning disabilities, and a space of solitude. Josh's involvement in his care allows for his assessment of the situation to register, if not prevail.

### 5.4 Moment Four—Interruptions

Having addressed three moments of successfully realised allyship in healthcare with people with learning disabilities, I turn to a moment that illustrates both a clinician's humanising practice and the restrictions under which they operate, and which limit the extent to which they can enact allyship with patients with learning disabilities.

Deb is a middle‐aged woman with learning disabilities. Deb requires psychotherapy but before she can be referred, she has to demonstrate that she possesses sufficient levels of comprehension to engage in therapy. To that end she has been referred to Olivia, a clinical psychologist. The assessment takes place in the reception room of a day centre, a space lacking the kind of privacy usually associated with a healthcare appointments. The assessment entails Olivia showing Deb images from the British Picture Vocabulary Scale. Beginning with fairly unproblematic ones, the images Deb has to recognise increase in complexity and abstraction. The images also demand precise description so that Deb’s reading of an expression as ‘surprized’, rather than ‘perplexed’ is registered as a mistake. I notice that I feel uncomfortable watching Deb ‘fail’ and small changes in Olivia’s body language make me believe that she is similarly invested in Deb completing the task successfully. Throughout the assessment, Olivia remains solidly present and attentive to Deb. At times Deb exhales forcefully and tilts her head back—a movement I interpret as a sign of frustration. When this happens, Olivia slows pauses and waits. On a couple of occasions she, using a soft tone of voice, asks Deb if she is ok to continue. This appears to reassure Deb sufficiently for her to continue with the assessment. Throughout the meeting, we can be seen by people moving through the car park. Indeed, while the doors are locked, Deb and Olivia are interrupted twice as people ring the bell, wanting to enter the room, one time to deliver a parcel. Although Olivia seems annoyed by the interruptions and apologises to Deb, she doesn’t appear able to stop them. Instead she continues the assessment.

Deb's appointment indicates that neither specialist services nor the skills of empathic clinicians such as Olivia can affect the broader structural disadvantage of people with learning disabilities. To illustrate, learning disability services often lack a base, with clinicians meeting patients in community spaces. Although such mobility enables patients to receive care without needing to travel or leave their familiar environment, it also has to contend with the disadvantage that community spaces are not always suited for medical appointments. This situation is closely related to Austerity, insofar as the reduction in public spending has led to a gradual loss of council‐run infrastructures that could previously accommodate multi‐disciplinary interventions into health and social care, such as learning disability services (Macpherson et al. 2023). Consequently, examples such as Deb's highlight the need for allyship to be enacted not only in interpersonal but also political arenas.

Similarly, although Olivia's practice is one of attentive kindness, it cannot transform psychology as a field that individualises and pathologises disability (Goodley et al. 2017). To illustrate, the images' increase in complexity reaches a form of social constructionist absurd when Deb's reading of ‘surprized’, rather than ‘perplexed’ is registered as a mistake. In this context, Olivia's kind attentiveness and ability to reassure Deb reminds me of how some empathic clinicians succeed in lessening the fear and pain that often accompany a medical encounter. That this encounter, itself is an instrument for the social construction of learning disability, necessitates similar capacities, gestures—somewhat uncomfortably—to how medicine flattens the distinction between a broken bone, and the cognition that it, through its very own discourses, defines as ‘inadequate’.

## 6 Conclusion

Learning disability allyship in healthcare must seek to transform the harmful disablist practices that have historically rendered patients with learning disabilities passive recipients of care whose purpose, focus and motivations they may not understand, much less be permitted to affect. Such transformations necessarily entail transformations of identities and roles, emphasising respect, attentiveness and a commitment to collaborative action. The here discussed data from Humanising Healthcare suggest that such transformations commonly coalesce around the patients acting, alongside clinicians, as one of the experts on their healthcare. Indeed, as Moment Three shows, such emphasis on the patient as an expert can position them as in control of aspects of their care. Although such control is, as discussed, limited by both structural conditions and ontological tensions between disability allyship and medicine, it is nevertheless a mechanism through which patients can influence their healthcare. As importantly, their becoming part of the team of experts can subvert clinicians' medical gaze and transform healthcare spaces into spaces where disability cultures are acknowledged, valued and facilitated.

As I highlighted, such changes require resources to ensure that patients are sufficiently informed to meaningfully engage. This, in turn, may call on clinicians to transform their communicative practices. Taken together such approaches could lead to healthcare settings becoming locations of disability culture, that is spaces that are committed to challenging ableism and attendant technologies of social disablement, for example, incomprehensible information or disabling views on autonomy as an individual, rather than relational, affair. However, areas of incommensurability remain as aspects of medicine—being themselves technologies of social disablement—are in conflict with allyship as an emancipatory practice. Although significant, such limitations should not serve to distract from the role clinicians can play in both advocating for patients with learning disabilities and enacting disability culture in the healthcare context. Indeed, although the data presented here were derived from encounters with people using verbal communication, transformative allyship with its focus on dialogue and enactment of disability culture can provide a useful point of departure into disability allyship in relation to patients who communicate non‐verbally or have profound/multiple disabilities. More research in this area is much needed.

## Author Contributions

**Bojana Daw Srdanovic:** conceptualisation, formal analysis, investigation, project administration, writing – original draft, writing – review and editing.

## Ethics Statement

The research underlying this paper has received ethics approval from the University of Sheffield.

## Conflicts of Interest

The author declares no conflicts of interest.

## Data Availability

The Humanising Healthcare study is, at the time of publication, an ongoing study. Data archiving will be completed at the end of the project and is likely to be partial to adhere to our ethical standards and safeguard participants. Please contact the author to discuss potential data sharing.
